# Food Addiction Screening, Diagnosis and Treatment: A Protocol for Residential Treatment of Eating Disorders, Substance Use Disorders and Trauma-Related Psychiatric Comorbidity

**DOI:** 10.3390/nu16132019

**Published:** 2024-06-26

**Authors:** Kimberly Dennis, Sydney Barrera, Nikki Bishop, Cindy Nguyen, Timothy D. Brewerton

**Affiliations:** 1 Department of Psychiatry, University of Illinois Chicago College of Medicine, Chicago, IL 60612, USA; drkim@suncloudhealth.com; 2 SunCloud Health, Chicago, IL 60062, USA; sydney@suncloudhealth.com (S.B.); nbishop@suncloudhealth.com (N.B.); cnguyen@suncloudhealth.com (C.N.); 3 Department of Psychiatry and Behavioral Sciences, Medical University of South Carolina, Charleston, SC 29425, USA

**Keywords:** food addiction, eating disorders, substance use disorders, nutritional rehabilitation, ultra-processed food, treatment, comorbidity, multi-morbidity

## Abstract

Food addiction, or ultra-processed food addiction (UPFA), has emerged as a reliable and validated clinical entity that is especially common in individuals seeking treatment for eating disorders (EDs), substance use disorders (SUDs) and co-occurring psychiatric disorders (including mood, anxiety and trauma-related disorders). The clinical science of UPFA has relied on the development and proven reliability of the Yale Food Addiction Scale (YFAS), or subsequent versions, e.g., the modified YFAS 2.0 (mYFAS2.0), as well as neurobiological advances in understanding hedonic eating. Despite its emergence as a valid and reliable clinical entity with important clinical implications, the best treatment approaches remain elusive. To address this gap, we have developed and described a standardized assessment and treatment protocol for patients being treated in a residential program serving patients with psychiatric multi-morbidity. Patients who meet mYFAS2.0 criteria are offered one of three possible approaches: (1) treatment as usual (TAU), using standard ED treatment dietary approaches; (2) harm reduction (HR), offering support in decreasing consumption of all UPFs or particular identified UPFs; and (3) abstinence-based (AB), offering support in abstaining completely from UPFs or particular UPFs. Changes in mYFAS2.0 scores and other clinical measures of common psychiatric comorbidities are compared between admission and discharge.

## 1. Introduction

The description of food addiction (FA) in humans by Gearhardt and colleagues in 2009 followed a series of studies in animals demonstrating the hedonic and addictive nature of highly palatable foods [1,2,3]. Since the measurement of FA in humans by means of the Yale Food Addiction Scale (YFAS) and its subsequent versions, an explosion of studies have substantiated its validity and delineated its phenomenology [4,5,6,7,8,9,10,11]. Multiple epidemiological studies indicate that FA, which has also become known as ultra-processed food addiction (UPFA), is quite common, and occurs in as many as 20% of the general population [8,12,13]. Although UPFA occurs in a large percentage of people who are not captured by traditional ED diagnoses, it often, but not always, co-occurs with EDs, particularly binge-type EDs (AN-BP, BN, BED, some types of OSFED) [8,14]. UPFA is also known to be associated with several psychiatric and non-psychiatric medical conditions, which have important etiological, developmental, treatment, prevention and social policy implications for patients who meet the criteria [15,16,17,18,19,20]. UPFA is an important clinical concept that may be used as a “proxy measure” for a matrix of interrelated clinical features, including greater ED severity, higher BMI, more severe trauma histories, greater symptoms of posttraumatic stress disorder (PTSD), greater psychiatric comorbidity and greater medical morbidity and mortality [20,21,22].

In addition, YFAS scores have been significantly correlated with higher negative affect, higher emotion dysregulation, higher eating disorder related psychopathology and lower self-esteem [21,22,23,24]. Higher scores on the YFAS are also related to an earlier age of first being at a higher weight and first dieting onset. YFAS scores are also significant predictors of objective binge eating frequency, above and beyond other measures [22].

Other research has shown that FA/UPFA is highly correlated with PTSD, food insecurity and acculturative stress in minoritized racial groups [25]. Access to a variety of foods, including UPFs and whole foods, falls along racial lines, with BIPOC (black, indigenous, and people of color) individuals being disproportionately more likely to live in food deserts, food swamps and food mirages with limited access to whole foods. It is important to note that the overwhelming majority of eating disorder research has focused on white females with restrictive eating phenotypes, and standard, evidence-based psychological and nutritional approaches to eating disorder treatment may not be generalizable to all patients with eating disorders (particularly those in historically marginalized racial and ethnic groups and those with clinically significant food addiction).

Despite these advances, FA/UPFA remains a relatively new and still controversial condition in clinical mental health care, and the most effective way to treat it, particularly from a nutritional perspective, represents a large gap in our evidence base [15,17,26,27,28,29,30,31,32,33,34,35,36]. To date, there has been only one randomized controlled trial of a dietician-led telehealth intervention that showed significant positive effects on addictive eating in community participants [37]. As a result of the above factors, we endeavored to develop a rational, scientifically based protocol for the assessment, diagnosis and treatment of individuals admitted to a residential program specializing in eating disorders, substance use disorders and co-occurring conditions.

Aims: Our hypothesis is that those diagnosed with UPFA will fare better with a harm reduction (HR) or an abstinence-based (AB) nutritional intervention compared to those who choose treatment as usual (TAU). This nutrition therapy approach is part of a larger overarching study examining the effectiveness of the entire treatment program on various outcome measures, which are described below. 

## 2. Materials and Methods

### 2.1. Participants

Inclusion criteria: Participants who are invited into the study include all patients admitted to a residential treatment program at SunCloud Health (SCH). Adults unable to consent, children and adolescents below the age of 18 years, pregnant women and prisoners are not involved in the study. Otherwise, there are no exclusion criteria. No individuals who are considered a member of any vulnerable population can take part in the study. This is a minimal-risk study, as it simply entails allowing us to analyze the data that we are already collecting for clinical treatment purposes for research purposes.

### 2.2. Setting and Resources

The study takes place in any and all SCH programs, including the residential treatment center (RTC), partial hospitalization program (PHP) and intensive outpatient program (IOP) levels of care. However, all subjects taking place in this arm of the study are patients who are initially admitted to one of SCH’s RTC programs. All patients complete assessment instruments onsite within 48–72 h of admission, and no collection of research data is conducted outside of SCH.

The time devoted to conducting and completing the research includes the time needed for collecting consent from patients, along with supporting the patients in answering any questions or concerns that they might have regarding participating in the research throughout their participation. Furthermore, the Research Coordinator or Data Manager follows up with patients regarding their participation in the research and completion of the 6-month and 12-month follow-up questionnaires. All persons assisting with the research have undergone training so that they are adequately informed about the study plan (protocol), the research procedures, and their duties and functions. Medical and psychological resources that subjects might need because of anticipated consequences of human research are readily available on site.

### 2.3. Research Plan and IRB Approval

The length of the study included patients’ time in treatment and one year after the date of discharge from all SCH programs. All patients who admitted to a SCH program were asked for consent to participate in this study. Therefore, it was anticipated that approximately 500–1000 participants each year would participate, ranging in age from 18 years old to 75 years old. Participants were only be recruited from groups who had already been admitted to treatment at one of our treatment centers, and there were no separate participant recruitment efforts.

The duration of an individual subject’s participation in the study included the total time in treatment and one year after the date of discharge from all SCH programs. Enrollment was ongoing and open-ended. The estimated date for the investigators to complete the study was from 3 to 5 years.

The assessment, follow-up and outcome portions of this study were approved by the Brany Institutional Research Board (File # 23-12-396-1474). Changes in mYFAS2.0 scores and other clinical measures of common psychiatric comorbidities and/or symptoms, i.e., EDs, SUDs, PTSD, major depression (MD), state-trait anxiety and quality of life (QOL), were compared between admission and discharge. These are described in detail below.

### 2.4. Variables, Measures and Diagnoses

#### 2.4.1. General Overview

After consent was obtained, the participant’s medical record and self-report questionnaire data collected over the course of treatment were de-identified, aggregated and analyzed. In addition, participants were also asked to complete self-report questionnaires 6 and 12 months after being discharged from our centers, and we obtained a release of information to collect height and weight data from the post-discharge providers. As such, the endpoints for data collection were as follows: admission to treatment, discharge from treatment, 6 months post-discharge and 12 months post-discharge. Questionnaire data were collected via RRDD or Owl Survey Software, which protect data using the best industry standards. Details on measures and procedures are provided below.

#### 2.4.2. Medical Record Data

The following data points were pulled from the medical record to be analyzed: date of birth; level of care admitted; specific program admitted; eating disorder diagnosis; substance use disorder diagnosis; posttraumatic stress disorder (ptsd) diagnosis; mood disorder diagnosis; other comorbid diagnoses; admission weight; admission height; highest weight at current height; lowest weight at current height; history of self-harm; history of suicidal ideation; history of suicide attempts; length of stay; type of transfer (post-discharge); type of discharge; discharge weight; discharge height; sex assigned at birth; gender identity; race; and sexual orientation.

#### 2.4.3. Ultra-Processed Food Addiction (UPFA) Assessment

Currently, UPFA is defined by high scores on the modified Yale Food Addiction Scale 2.0 (mYFAS 2.0), which has well-established reliability and validity [13,38,39,40,41,42]. The modified Yale Food Addiction Scale 2.0 (mYFAS 2.0) is an abbreviated, 13-item version of the Yale Food Addiction Scale 2.0 (YFAS 2.0). The mYFAS 2.0 has one question to assess each of the 11 DSM-5 diagnostic criteria for substance use disorders (SUDs), plus two questions to assess clinically significant distress and impairment [43]. The mYFAS 2.0 performs similarly on psychometric indicators to the full versions of the scale and is a useful brief assessment tool for UPFA [39,43].

A FA/UPFA diagnosis using the mYFAS 2.0 is based on DSM-5-TR criteria for a SUD [44]. In this study, all treatment team staff worked with medical providers on using the accurate ICD diagnosis code (F19.20 other psychoactive substance dependence) for anyone diagnosed with FA/UPFA as part of their overall clinical picture. SUDs span a wide variety of problems arising from substance use and cover 11 widely accepted criteria [45]:

1. Taking the substance in larger amounts or for longer than one meant to;
2. Wanting to cut down or stop using the substance but not managing to;
3. Spending a lot of time getting, using, or recovering from use of the substance;
4. Cravings and urges to use the substance;
5. Not managing to do what one should at work, home, or school because of substance use;
6. Continuing to use, even when it causes problems in relationships;
7. Giving up important social, occupational, or recreational activities because of substance use;
8. Using substances again and again, even when it puts you in danger;
9. Continuing to use, even when one knows one has a physical or psychological problem that could have been caused or made worse by the substance;
10. Needing more of the substance to get the effect one wants (tolerance);
11. Development of withdrawal symptoms, which can be relieved by taking more of the substance.

The 11 criteria outlined in the DSM-5-TR can be grouped into 4 primary categories: physical dependence, risky use, social problems, and impaired control. In addition, the DSM-5-TR allows clinicians to specify how severe or how much of a problem the SUD is, depending on how many symptoms are identified. The mYFAS 2.0 follows the exact same guidelines for mild (2–3 symptoms), moderate (4–5 symptoms) and severe FA (6 or more symptoms) [45].

“Secondary” food addiction (or “false positives”) can occur if the test is administered to people in a state of malnourishment, which may drive a subjective sense of craving and perceived loss of control of eating, since approximately 50% of patients with anorexia nervosa restricting type (AN-R) have been reported to meet FA/UPFA criteria [15,46,47]. Nevertheless, any and all suspected “false positives” should be reviewed and discussed with the patient, and the test should be readministered after a period of observed consistent nourishment. Secondary food addiction due to severe dietary restriction or malnourishment is expected to be negative upon retesting in a nourished state in the majority of patients. If, after a period of observed nourishment, the patient still tests positive and does not meet the DSM-5-TR criteria for AN-R, this indicates clinically significant FA/UPFA.

As previously stated, secondary food addiction (or “false positives”) can occur, primarily in undernourished patients with restrictive AN and over control as the predominant ED behavioral symptoms. Dietary restraint as a primary symptom of a restrictive eating disorder (pathological restraint) must be distinguished from dietary restraint related to overfullness or GI distress related to OBE (objective binge episodes) and/or decreased consumption in the 8–24 h after consuming large amounts of food in OBEs. It also is distinguished from adaptive dietary restraint (which we see, for example, when people avoid foods they have allergies to, food sensitivities to, or medical conditions which worsen with consumption of those foods, e.g., Celiac, migraine, ADHD) [36]. Following an eating plan that is low in exposure to UPFs can be conceptualized as adaptive dietary restraint for some people with FA/UPFA, and should not be pathologized or misconstrued as an indicator that the patient has succumbed to a restrictive ED or diet culture. More selective eating is NOT the same as caloric restriction. Furthermore, choosing to follow a low-exposure meal plan does not necessarily mean the patient will lose weight. A low-exposure meal plan can be distinguished in this way from a diet. In fact, low-exposure meal plans are compatible with weight restoration for those with FA/UPFA who clinically need them. FA/UPFA diagnosis and treatment are inherently weight-neutral and compatible with a weight inclusive overall treatment approach. It is important to remember that UPFA occurs in people of a broad range of body sizes, including those within the BMI range presently considered to be normal weight.

#### 2.4.4. Assessment of Comorbid Symptoms

All mYFAS 2.0 scores were considered in the context of their full diagnostic picture, including scores on other assessments, which were administered within 48–72 h of admission [15,17]. These included the following self-report screening instruments, all of which were found to have good reliability and validity:

- The Patient Health Questionnaire-9 (PHQ-9) is a 9-item self-report instrument that screens for symptoms of major depression (MD) during the past 2 weeks. A PHQ-9 score ≥10 has a sensitivity of 88% and a specificity of 88% for major depressive disorder. PHQ-9 scores of 5, 10, 15 and 20 signify mild, moderate, moderately severe and severe depression, respectively [48].
- The short form of the Spielberger State Trait Anxiety Inventory (STAI) is a 10-item self-report measure that assesses state and trait anxiety symptoms. It is highly correlated to the full form of the STAI and has been shown to have satisfactory reliability and validity [49,50]. The cut-off score for the state scale is >9.5, while the cut-off for the trait scale is >13.5 [51,52,53].
- The Eating Disorder Examination Questionnaire (EDEQ) is a 28-item self-report instrument that assesses ED symptomatology during the prior 28 days. The EDEQ has a mean global scale for an overall assessment of ED symptoms, which consists of 4 separate subscales in the domains of restraint, eating concern, weight concern and shape concern [54]. Norms for adults from various populations have been published and will be utilized in interpreting scores [55,56,57,58,59,60].
- The Life Events Checklist for DSM-5 (LEC-5) is a self-report instrument that assesses for 17 possible PTSD criterion A traumatic events. Patients who endorse a life-threatening event or sexual assault that happened to the individual and/or was witnessed, together with patient responses on the PTSD Checklist (see below), qualify for a presumptive DSM-5 diagnosis of PTSD [61,62].
- The PTSD Checklist (PCL-5) is a 20-item self-report measure that assesses for DSM-5 cluster B, C, D and E symptoms of PTSD over the past month [63]. A total cutoff score of ≥33, plus meeting DSM-5 criteria B-E, are used as reliable indicators of provisional PTSD in other studies [64,65,66].
- The International Trauma Questionnaire (ITQ) is an 18-item self-report instrument that assesses for ICD-11-defined symptoms of both PTSD and complex PTSD [67,68]. Numerous studies have shown support for the factorial and discriminant validity of both PTSD and complex PTSD ICD-11 diagnoses [69].
- The Dissociative Subtype of PTSD Scale (DSPS) is a 15-item self-report instrument that assesses for symptoms of DSM-5 PTSD dissociative subtype. Factor analyses support three factors, reflecting derealization/depersonalization, loss of awareness and psychogenic amnesia [70,71].
- The Alcohol Use Disorder Identification Test (AUDIT) is a 10-item self-report screen that assesses for symptoms of alcohol use disorder (AUD) [72]. Cut-off scores of 7 and 8 show good sensitivity and specificity, respectively [73].
- The Drug Abuse Screening Test (DAST-10) is a 10-item self-report screening tool that assesses for symptoms of substance use disorders (SUDs) other than alcohol and tobacco. A cut-off score of 2 shows good sensitivity and specificity [73,74].
- The World Health Organization Quality of Life abbreviated scale (WHOQOL-BREF) is a 26-item self-report measure of an individual’s quality of life in 4 separate domains: physical health, psychological, social relations and environment [75,76]. Confirmatory factor analysis has shown that the WHOQOL-BREF has good to excellent psychometric properties of reliability and validity [75].

#### 2.4.5. Patient Education, Collaboration and Treatment Approach

As noted above, it is imperative to talk with patients about their test results, including their scores on mYFAS 2.0 and their interpretations. Full transparency is a foundation of trauma-informed care and helps to build rapport and trust. Results should be shared with the patient in the context of initiating a discussion about the extent to which their symptoms can be framed using a FA/UPFA perspective. *An important distinction to be made clinically is that a diagnosis of FA/UPFA based on mYFAS2.0 does not automatically determine treatment.* Diagnosis of FA/UPFA is well established, and it is a reliable and valid concept that is supported by a large body of scientific work in both animals and humans. The best treatment for FA/UPFA, on the other hand, is not well researched or established, and much work remains to be carried out to determine what the best approaches are, particularly when comorbid with an ED. SCH is uniquely equipped to study this and to lead in the development of patient-centered treatment modalities. Evidence-based treatments (EBT) certainly rely on the ever-growing body of systematic scientific research knowledge, particularly from clinical trials, but this is just one leg of a three-legged stool that defines EBT [77]. The other two equally important legs are clinician experience and patient preferences. Therefore, it is important to explore what the patient’s experiences have been in the past. Do they accept the diagnosis of FA? What strategies have they employed in the past for loss-of-control eating? What has worked and what has not? Have they ever employed a harm reduction strategy or an abstinence strategy, and if so, what were their effects? Notably, there have been *no controlled treatment trials yet for FA/UPFA in residential care*, and established treatments for ED can be helpful for some, but not all. Part of the mission of SunCloud is to explore what strategies work well for which patients with FA.

Treatment can progress using the tools and principles that have already been established, especially when considering the close relationship of FA/UPFA to EDs, SUDs, PTSD and other psychiatric comorbidities. Cognitive-behavioral therapies (CBTs) are well established evidence-based therapies for all of these disorders, and they can readily be applied to FA/UPFA. It is also known that principles of harm reduction are very useful in the treatment of SUDs and related disorders [78]. Establishing safety and reducing harmful behaviors are tried and true approaches, and also include second-generation techniques such as DBT skills training and a variety of trauma-focused treatments [79]. The principles of harm reduction include: commitment to evidence and social justice; practical, flexible and patient-centered goals; highly individualized care; focus on improving QOL (values defined by patient); commitment to respecting patient choice; avoiding adding to stigma or discrimination; taking a collaborative, non-judgmental and non-coercive approach; and meeting patients where they are [80].

#### 2.4.6. Treatment Options and Informed Consent

Once informed consent is signed and the FA/UPFA protocol is initiated, the next step consists of the following treatment components.

- Psychoeducation: An integral part of the treatment protocol is psychoeducation (including myth busting for those who have had ED treatment, e.g., all foods do not fit for all people, there are nutrition sensitive medical conditions, etc.).
- Psychopharmacology: An integral part of the treatment protocol is psychotropic medication management of multi-morbidity diagnoses as well as the use of neuromodulation therapies when indicated.
- Medical Monitoring: Part of the treatment protocol is medication management and monitoring of medical multi-morbidity when present.
- Movement therapy: Part of the treatment protocol is developing an individualized meaningful and pleasurable movement plan with RD.
- Nutrition therapy: integrating the above concepts as they relate to FA/UPFA.
- Engaging with a recovery peer support community: Principles from 12-step facilitation (TSF, an evidenced based treatment for SUDs) are used to facilitate client examination of cognitive distortions; initiation of behavioral activation; redefining meaning and purpose in relation to self, others and the universe; and connection to recovery community.
- Trauma-focused treatments: FA/UPFA is strongly linked to trauma histories, PTSD and its symptoms [16,17,18,81,82,83,84]. Therefore, addressing PTSD symptoms, as well as related comorbidities, is an important and probably necessary component of the comprehensive treatment of FA/UPFA.
- The aim of SCH is to have a weight-neutral, size-inclusive, *adaptively intuitive* eating plan and nutritional intervention protocol that goes into place along with it. Meal plans are patient-specific and aim to help patients with food behaviors and/or food types identified by them as problematic (specific eating behaviors and/or foods which regularly diminish their capacity to exercise adaptive dietary restraint aligned with their authentic values).
- Meal plan interventions can be broadly grouped into the following approaches:
  ○ **Treatment as usual (TAU)**: standard eating disorder dietary approaches;
  ○ **Harm reduction (HR)**: support in decreasing all UPFs from current percent of one’s meal plan to a lower percent of the overall plan, or decreasing the amount of consumption of particular identified UPFs;
  ○ **Abstinence-based (AB)**: support in abstaining completely from certain food substances (ex., added sugars, high carb/high fat combination foods (hyperpalatable or UPFs).
- Meal plans in recovery are dynamic and are meant to change with the person as their brains change in recovery and over the span of their lives. Exposure to foods that have been identified as problematic should be implemented with therapeutic and group support, keeping in mind that setting and context can influence a person’s reaction to foods (e.g., “I am not triggered to want more after eating a Chips Ahoy cookie at meal support table in RTC where extra food is locked in the kitchen, and I am surrounded by support. At home alone with a whole bag of Chips Ahoy in my cabinet, I can’t stop eating them.”).
- Meal plans are calorie-replete and include all food groups or macronutrients.
- Goal weight ranges and caloric energy needs are estimated by the registered dietitian after nutrition assessment using evidence-based methods that consider weight history, growth charts, genetic and familial factors, dieting history, current eating patterns and patient preference. It is important to account for medications the patient is taking that may impact appetite and/or weight in one direction or the other.
- For people who wish to try an AB intervention, the initial guiding principal for meal plans would not include any foods with added sugars under the Ingredients List on food labels, or would include a meal plan with little to no NOVA-4 foods. NOVA-4 foods have the highest degree of processing and include packaged formulations of substances developed in laboratories that contain very little, if any, whole foods [85,86,87,88].
- An HR intervention may be creating a red/yellow/green food list and starting with decreasing the amount of red foods or abstaining from them. Specific foods would be identified as problematic per the patient and categorized as **red** (always problematic), **yellow **(sometimes problematic), or **green **(never problematic).
- Exposure-based therapies are an important component of each of the treatment approaches described (TAU, HR, AB). Specific interventions vary based on patient preference and clinical characteristics at any given time in the course of care [89,90,91,92,93].
- It is important to understand that the amount of psychosocial support that a person receives in a 24/7 residential setting surrounded by peers without access to excess food may impact their subjective reports of cravings and can improve inhibitory control when full. In deciding the type of meal plan to be employed for any given patient, it is important to consider a person’s lived experience of what happens in their home setting with free access to food and less psychosocial eating support.

#### 2.4.7. Statistics

- Procedure for Statistical Analysis: Data are de-identified, and a record number (rather than name or any other identifying information) is used. The data are then aggregated and analyzed. In terms of sample size determination, the study is ongoing in nature, so data from all participants are analyzed. The sample size is determined by the number of patients admitted to any of our centers throughout the duration of the study.
- Statistical Methods. Both descriptive and inferential statistical procedures are used to explore research questions related to symptom change in treatment and effectiveness of treatment after discharge. The data are analyzed throughout the duration of the study based on the type of nutritional intervention employed. A *p*-value of .05 is used as the level of significance criteria, but this is corrected for the number of analyses performed. Missing data are dealt with using the expected maximization (EM) technique with a cutoff of 20%. All eligible participants who have consented to the study and who have provided data are used in the analysis. For analyses of change from admission to discharge, dependent variables of interest are compared using multivariate analyses of variance (MANOVA), with the type of nutritional intervention as the independent or fixed variable. Dependent variables include all change scores measured between admission and discharge, including the mYFAS2.0 total score, the EDEQ Global score, the PHQ-9 score, the PCL-5 total score, the ITQ PTSD score, the ITQ DSO score, the STAIs state score, the STAIs trait score, the DPTSD score, the AUDIT score, the DAST-10 score and the WHOQOL-BREF total and domain scores. Several variables are used as covariates in multivariate analyses of covariance (MANOVA), including age, admit BMI and baseline admission scores for all psychometric measures. The number of days spent in RT (length of stay, LOS) is used as a weighting factor in all MANOVA analyses. Missing data are not included. All statistical analyses are performed using general linear model multivariate analyses in SPSS 28 (IBM, 2021). Effect sizes are calculated as partial Eta squared (ηp2). Least squares differences (LSD) are used for post hoc comparisons. For comparisons between admission, discharge and 6- and 12-month follow-up data, linear mixed multilevel models (MLM) are utilized as the primary statistical method of analysis. MLMs are accommodating of missing data at various data points collected at differing spaced time points without relying on imputation of data. MLMs also allow control of various baseline covariates [94,95,96,97,98].

## 3. Results

To date, there have been 165 patients admitted to SCH residential care for psychiatric treatment since November 2023. The demographic and clinical characteristics of these patients, including age, admit BMI, length of stay, sex assigned at birth, gender orientation, sexual orientation, race/ethnicity and diagnoses, are shown in Table 1. The majority of patients admitted have SUDs, mood disorders, and/or PTSD. At least one-third also meet the criteria for UPFA, and 40% have EDS.

Of these 165 patients, 54 (33%) met mYFAS 2.0 criteria for FA/UPFA. There were no differences in age between those with and without FA/UPFA, but admission BMI was significantly higher in those with FA/UPFA (30.7 ± 10.0) compared to those without (25.8 ± 5.1, *p* ≤ .001, F = 32.0).

Of the 54 patients admitted with FA/UPFA, 32 (72%) had an admission diagnosis of an ED, and conversely, 39 (60%) of the 65 ED patients admitted met the criteria for FA/UPFA.

## 4. Discussion

There has been an explosion of research into the phenomenon of FA/UPFA over the last decade, which has in large part been due to the development and established reliability of the YFAS and its permutations. FA/UPFA has been reported to commonly co-occur in patients with EDs, and our initial findings are compatible with this, yet there is a paucity of research on how best to treat FA/UPFA, particularly in those with EDs. Many clinicians in the ED field assume that a diagnosis of FA/UPFA implies an abstinence model of treatment, which is all too often conflated with dietary restriction and/or diet culture. This occurs despite the emergence of a wealth of data showing that UPFs are harmful as well as potentially addictive [36]. This fact notwithstanding, the best approach for individuals with addictive eating remains to be established, particularly in higher levels of care and in patients with EDs.

This protocol provides a model for an evidence-based treatment approach that can be individually tailored to a patient’s history, course of illness, and personal preferences [77]. Specifically, three major nutritional approaches are offered and studied in those with FA/UPFA, including TAU, a HR approach and an AB approach. Repeated assessments completed at discharge and then again at 6 and 12 months of follow-up provide useful information about how best to treat individuals with FA with and without an ED and/or SUD. In addition to offering nutritional rehabilitation, evaluation and integrated treatment of comorbid disorders (such as medical conditions, PTSD, EDs, SUDs and mood disorders) are also implemented.

## 5. Conclusions

A major limitation of this protocol is that it is not a randomized, controlled trial, which would be impossible to conduct in a treatment setting. Nevertheless, we believe that the approach that we have outlined is not only scientifically sound, but clinically practical and a reflection of the current state of the art.

Another limitation of our study is the potential for selection bias. Recruiting patients solely for our treatment programs likely introduces selection bias and therefore limits the generalizability of our findings. We also acknowledge that patients from underprivileged, marginalized communities are underrepresented in our sample despite being in a population that is preferentially targeted by UPF advertising [99]. In addition, they may have limited access to whole, plant-based foods and may be subject to food insecurity [25,35,100]. Given this, our program staff will strive to provide culturally attuned, comprehensive care. In addition, demographic features will be controlled for and their influence noted when reporting our future results.

Furthermore, our patient population does not include publicly insured individuals, which may limit the generalizability of our findings. Finally, the study’s longitudinal design with follow-up measures at 6 and 12 months after discharge may be susceptible to attrition bias, which could affect the validity of the findings.

## Figures and Tables

**Table 1:** Demographic and clinical features of a sample of patients admitted to residential treatment.

Mean (±SD) Age: 31.4 ± 11.6 years
Mean (±SD) Admission BMI: 28.1 ± 7.6 kg/m^2^
Mean Length of Stay: 31 days (range of 5–99 days)
Sex Assigned at Birth:
Female: 62.5%
Male: 37.5%
Gender Identity:
Female: 53.2%
Male: 34.5%
Non-binary: 6.0%
Transmale: 3.6%
Transfemale: 1.6%
Sexual Orientation:
Heterosexual: 44.1%
Queer: 35.9%
Bisexual: 6.5%
Pansexual: 1.2%
Asexual: 0.8%
Gay: 1.8%
Lesbian: 0.6%
Missing: 9.4%
Race/Ethnicity:
White: 77.1%
Black/African-American: 4.8%
Hispanic/Latino: 5.3%
Asian: 4.1%
Multi-racial: 7.3%
Missing: 1.8%
Diagnoses:
Ultra-processed food addiction: 33%
Eating Disorder: 40%
PTSD: 59%
Substance-related or addictive disorder: 74%
Mood disorder: 77%

## Data Availability

The data presented in this article are not readily available because they are part of an ongoing study.
